# Community unit performance: factors associated with childhood diarrhea and appropriate treatment in Nyanza Province, Kenya

**DOI:** 10.1186/s12889-017-4107-0

**Published:** 2017-02-16

**Authors:** Yoshito Kawakatsu, Junichi Tanaka, Kazuya Ogawa, Kenneth Ogendo, Sumihisa Honda

**Affiliations:** 1 Graduate School of Biomedical Sciences, Nagasaki University, 1-7-1 Sakamoto, Nagasaki, Japan; 2 Strengthening Pro-Poor Community Health Project, Lagos, Nigeria; 3 Ministry of Health, Nairobi, Kenya

**Keywords:** Community health worker, Infectious disease, Diarrhea, Health-seeking behavior, Multi-level analysis, Kenya

## Abstract

**Background:**

The government of Kenya launched its community health strategy in 2006 to improve certain aspects of its community health program. Under the strategy, community units (CUs) were established as level one of the Kenyan health system. A core member at this level is the community health worker (CHW). The objective of this study was to assess the relationship among the performance of the CUs, the prevalence of childhood diarrhea and appropriate treatment for it by controlling individual and community-level factors.

**Methods:**

The main dataset used in this study was the 2011 Nyanza Province county-based Multiple Indicator Cluster Survey (MICS). In addition, based on the list of community units in Nyanza Province, Kenya, we identified the area’s CUs and their performance. MICS data and data on CUs were merged using sub-location names. There were 17 individual and two community-level independent variables in this study. Bivariate analysis and a multilevel logistic regression were performed.

**Results:**

Factors significantly associated with a lower prevalence of diarrhea among children under five were the child’s increasing age, middle-aged household heads, children who received more attention, water treatment and rural versus urban area residence, while male children and highly performing CUs were significantly associated with a higher prevalence of diarrhea. In addition, middle wealth index, severity of diarrhea and middle- and high-CU performance were significantly associated with appropriate treatment for childhood diarrhea.

**Conclusions:**

Although this study found that children living in areas of high CU performance were more likely to have diarrhea, these areas would have been identified as being more at risk for diarrhea prevalence and other health concerns, prioritized for the establishment of a CU and allocated more resources to improve the performance of CUs. A higher CU performance was significantly associated with the appropriate treatment. It was suggested that CHWs could have a positive effect on the community, as demonstrated and promoted by appropriate health-seeking behavior and treatment for childhood diarrhea.

## Background

In efforts to achieve universal health coverage (UHC), a key challenge is an inadequate health workforce, especially in resource-poor countries [1]. To deliver essential health interventions to every community, community health workers can undertake a wide range of tasks [2]. It is recommended that investment in building CHW skills and the maintenance of their performance are beneficial to UHC [3].

To empower households and communities to improve their own health, the Kenyan Government developed and launched the Community Health Strategy (CHS) in 2006. The strategy was well articulated as one of the flagship programs in the National Health Sector Strategic Plan 2005–2010 (NHPP II) [4]. One of the main purposes of this strategy was to enhance access to health care services in order to improve productivity and thus reduce poverty, hunger, and child and maternal deaths. In this strategy, the community unit (CU) is defined as level one of the Kenyan Health System. It consists of a community health committee (CHC), two community health extension workers (CHEWs) and community health workers (CHWs).

The CHC is the governing body that coordinates all community health activity; its members are community leaders. There are two types of CHEWs in a CU. One is the facility CHEW, who works in a health facility. The other is the community CHEW, who works mostly at the community level. Both supervise the work of the CHWs. Among the groups, CHWs were the only direct service providers to community members.

In each CU, 50 community health workers (CHWs) were selected from the community between 2006 and 2010. The household coverage per CHW was 20. Later in 2010, the implementation strategy of CHS, especially distribution of personnel, was revised and about 10 CHWs were chosen after that. After the revision, the household coverage per CHW was 100 in Nyanza province. As a reward for increased household coverage per person, the government had planned to provide a small stipend. Although all CUs established both before and after the revision should have been standardized, based on the guidance of the Kenyan Ministry of Health (MoH), the progress of the implementation was varied, depending on the funds received from the government, international organizations, and NGOs. As a result, most CHWs were still volunteers in 2011, with the exception of the areas where particular partners paid the allowance. Although the training procedure for CHWs was standardized by the Kenyan MoH, their performance varied and was influenced by individual and contextual factors [5, 6].

Within this environment of increased attention to community health issues, the high burden of diarrhea amongst young children was an area of focus as diarrheal diseases were the second leading cause of mortality worldwide among children under five in 2008 [7]. Almost half of these deaths occurred in Africa [7]. Although numerous facility- and community-based interventions have been implemented to improve access to and uptake of effective preventions and treatments [8], the burden of diarrhea is still substantial in sub-Saharan countries. Therefore, prevention of diarrheal diseases was one of the focal areas of CHS in Kenya. The CHWs received the training, including methods for the prevention of diarrhea, water safety, sanitation and hygiene-related issues. According to the recent Kenyan Demographic Health Survey [9], 16.6% of children under five had experienced diarrheal diseases within the two weeks preceding the survey, and 2.6% had had severe diarrhea with blood. Among them, 13% had not received any treatment.

Although CHS was implemented from 2006, few studies have evaluated the impact of CUs and CHWs according to CHS [5, 6, 10]. Therefore, the objective of this study was to assess the performance of CUs and other individual- and community-level factors on the prevalence of diarrhea and its appropriate treatment among children under five in western Kenya.

## Methods

### Dataset

The main dataset used is this study was the Nyanza Province County-based Multiple Indicator Cluster Survey (MICS) of 2011. The detailed survey methodology is available in the Kenya, Nyanza Province Multiple Indicator Cluster Survey 2011 final report [11]. The dataset includes 30,439 household members from 6,828 households that participated in the survey. A structured questionnaire was administered to gather a wide range of information, such as socio-economic characteristics and health conditions. In addition, a list of CUs in Nyanza province, obtained from the Kenyan Ministry of Health, was utilized to identify the communities with CUs and their performance. The total number of CUs in Nyanza province of Kenya was 586. Both MICS data and data on CUs were merged using sub-location names.

### Measures

#### Outcome variables

There were two outcomes of this study. One was the number of cases of diarrhea among children under five years old within the two weeks preceding the survey. In the MICS survey, “Diarrhea is determined as perceived by mother or caretaker, or as three or more loose or watery stools per day, or blood in stool” [11]. Regardless of the severity of each case, we coded a value of 1 if children had had diarrheal symptoms during the two weeks preceding the survey, and a value of 0 if they had had no symptoms. The second outcome was the parent or caregiver’s treatment choice for the child’s diarrhea. Appropriate treatment for childhood diarrhea in this study, coded as 1, was defined as any of the following treatments: ORS, Zinc, intravenous fluids, or antibiotics for bloody diarrhea, while inappropriate behavior, coded as 0, was defined as no treatment, herbal medicine or ot her home remedy, antimotility, unknown pill, syrup and injection, or other.

### Independent variables

There were 17 individual and two community-level variables, shown in Table 1. Variables regarding the characteristics of household heads were included in the analysis since these were the people who made decisions or could influence the mother’s or caregiver’s decision making. Household wealth was calculated by a principal component analysis using the respondents’ household assets for the whole survey dataset. This index was used to capture socioeconomic status and was divided into five categories: poorest, poor, middle, rich and richest.

**Table 1:** Sample characteristics of children under five and their families MICS survey 2011 Nyanza, Kenya

Dependent variables	N (%)
Experienced diarrhea within the previous 2 weeks	
Yes	781 (15.8)
No	4174 (84.2)
Appropriate treatment for childhood diarrhea	
Inappropriate	481 (61.6)
Appropriate	291 (37.3)
Unknown	9 (1.2)
**Individual Variables for both analyses**	
Child’s gender	
Female	2441 (49.3)
Male	2514 (50.7)
Child’s age (years)	
< 1	969 (19.6)
1	863 (17.4)
2	1017 (20.5)
3	1093 (22.1)
4	1013 (20.4)
Relationship to household head	
Biological child	3912 (79.0)
Grandchild	872 (17.6)
Other	171 (3.5)
Household head’s gender	
Female	1236 (24.9)
Male	3719 (75.1)
Age group (years)	
< 30	1370 (27.7)
30–34	934 (18.9)
35–39	814 (16.4)
40–49	858 (17.3)
≥ 50	979 (19.8)
Education level	
No education/Primary level	3390 (68.4)
Secondary level or higher	1565 (31.6)
Household wealth index	
Poorer	1173 (23.7)
Poor	1037 (20.9)
Middle	1006 (20.3)
Rich	937 (18.9)
Richer	802 (16.2)
Number of household members	
5 or fewer than 5	2406 (48.6)
More than 5	2549 (51.4)
Number of children under five	
One	1553 (31.3)
More than 1	3402 (68.7)
Paying attention to the child	
Less attention	2780 (56.1)
More attention	2175 (43.9)
Playing with the child	
No playing	1012 (20.4)
Playing at least once within the past 3 days	3943 (79.6)
**Individual Variables for the first analysis on diarrhea cases**	
Water source for drinking	
Unimproved sources of drinking water	2445 (54.3)
Unprotected well	410 (8.3)
Unprotected spring	606 (12.2)
Tanker-truck	3 (0.1)
Cart with small tank/drum	14 (0.3)
Surface water	1412 (28.5)
Improved sources of drinking water	2510 (45.7)
Piped water	471 (9.5)
Protected well	311 (6.7)
Protected spring	971 (19.6)
Public tap/standpipe	246 (5.0)
Tubewell/borehole	247 (5.0)
Rainwater collection	244 (4.9)
Bottle water	0 (0.0)
Water treatment	
No treatment	2104 (42.5)
Inadequate treatment only	113 (2.3)
Adequate treatment	2738 (55.3)
Hand-washing facility	
Not available	4809 (97.1)
Available	146 (3.0)
Possession of refrigerator	
No	4868 (98.2)
Yes	87 (1.8)
**Individual Variables for the second analysis on appropriate treatment**	
Severity of diarrhea	
Without blood	673 (87.2)
With blood	99 (12.8)
Possession of at least one media device	
No	80 (10.4)
Yes	692 (89.6)
Community Variables for the two analyses	N (%)
Area	
Urban	505 (10.2)
Rural	4450 (89.8)
Performance of CHWs in the CU	N / CUs (%)
None/Poor (Number of CHWs and CUs)	2639/156 (53.3)
Middle	1483/91 (29.9)
High	833/51 (16.8)

Two binary variables were created: paying attention to the child and playing with the child. These variables indicated the degree of parental and family attention and care given to children. The variable ‘paying attention to the child’ was generated by the question: “Sometimes adults taking care of children have to leave the house to go shopping, wash clothes, or for other reasons and have to leave young children. On how many days in the past weeks was (name) left alone? or left in the care of another child (that is, someone less than 10 years old)?” [11]. The variable was classified into two groups: children who were paid less attention by family members and children who were paid more attention. That is, if the participants answered “one or more days left their children alone or only with other young children,” they were categorized as children who were paid less attention. If children always remained with adults, they were categorized as children who were paid more attention. As a result of being left alone or only with other young children, children are more at risk of suffering accidents [11]. ‘Playing with a child’ was created by using four questions as to whether or not household members had engaged in the following activities in the previous three days: reading books, taking the child outside, playing with the child, and naming, counting or drawing with the child. ‘Playing with a child’ was categorized as either playing with the child at least once in the past three days or not playing with the child at all during this time period.

Identifying the source for drinking water was generated from a question about the main source of drinking water for household members. Improved drinking water sources included piped water, protected wells, protected springs, tubewell or borehole, rainwater and bottled water [12]. This variable was coded as ‘improved’ or ‘unimproved’.

Water treatment was the methods to make water clean and safe to drink, which was categorized as “no treatment or inadequate” and “adequate treatment”. Adequate water treatment was defined as methods to disinfect water by killing harmful pathogens, such as boiling, adding bleach or chlorine, and using a water filter [12]. If one of these methods was used in the household, it was categorized as adequate treatment. Inadequate methods which were not sufficient to disinfect water included the choices; “strain it through a cloth” and “let it stand and settle” [12].

There were two additional variables for the analysis of appropriate treatment: possession of media devices and severity of diarrhea. Possession of media devices included radio, TV, mobile or landline phone, computer and the Internet. If a family had at least one of these devices, we grouped it as a household with at least a media device. Another variable, severe diarrhea, was defined as diarrhea with blood.

There were two community level variables: Area (rural or urban) and the performance of the CUs. The definition of rural and urban was provided in the 2009 Kenya Population and Housing Census [13]. The performance of the CUs was determined using three criteria developed by the Kenyan Ministry of Public Health and Sanitation (MoPHS). The first was the presence of minutes of monthly meetings, such as Dialogue Days and Action Days; the second was the presence of all key persons (CHC, CHWs, and CHEWs), all of whom must have finished the standardized training program, and the third was the presence of reporting tools for the community health information system (CHIS). Dialogue Day and Action Day meetings are held monthly with the CHC, CHWs, CHEWs and people from the community. In the Dialogue Day meeting, current health problems within the community and how to solve them are discussed. Action Day is a day to implement the solution decided on during the Dialogue Day meeting. There are two main official reporting tools in the CHIS: the CHW service delivery log book and the CHEW summary. After summarizing the CHW service delivery log book using the CHEW summary tool, the latter is submitted to the CHS focal person in a district followed by the CHS departments at provincial and national levels.

Each of these variables scored 1 if it they were available. If CUs received three points, they were considered “high performance CUs” because they had all required materials and functions based on the criteria. CUs with scores of 1 or 2 were categorized as “middle performance CUs.” CUs with a score of “0,” as well as areas without CUs, were regarded as reference groups, because the low performing CUs had no evidence of CU activity.

### Statistical analysis

A multilevel logistic regression analysis was performed in order to take account of the hierarchical structure of the data. This meant that individuals (level 1) were nested within communities (level 2). Multilevel analysis is a suitable approach to take into account the community level context, as well as individual characteristics. We computed three models in order to decide upon the most suitable final model. There was no independent variable in model 0, while only individual variables existed in model 1 and both individual and community variables were present in model 2. Based on the likelihood ratio test and the variance of random effects, we selected the final model with lower log likelihood and the variance of random effects. Both bivariate and multilevel logistic regression analysis with sample weights were performed using Stata 12 (Stata Corporation, College Station, TX).

## Results

Out of 300 Enumeration Areas (EAs) in the MICS survey, two were excluded because there was no information as to whether or not the CUs had been established. Therefore, the total number of EAs with full sets of CU data used in the analysis was 298. In this matched dataset, there were 5,045 children under five, but there were missing data for dependent and/or independent variables for 90 children. Thus the final sample included in this study was restricted to 4,955 children under five and their family members; this number was used to determine factors relating to childhood diarrhea. The data in Table 1 show that 19.6% of these children were under one year of age, and 79% were the biological children of the household heads. The percentages of participants who could access an improved drinking source and adequately treated their drinking water were 45.7% and 54.3% respectively. More than half (53.3%) lived in areas without CU or with the low-performing CU.

Out of 4,955 children, 781 (15.8%) had experienced diarrhea in the two weeks before the survey. However, treatment methods among nine of the children were unknown, so the total number of child participants with diarrhea included in the analysis for the appropriate treatment was 772 (98.8% of the original 781). In this sample, 12.8% of their diarrhea was bloody diarrhea, and 37.3% of them took appropriate treatment.

### Factors influencing the prevalence of childhood diarrhea

Table 2 shows the prevalence of diarrhea and appropriate treatment by independent variables and unadjusted odds ratios. The significant factors in multilevel analysis mentioned below were also significant in bivariate analysis.

**Table 2:** Prevalence of diarrhea and appropriate treatment by independent variables and unadjusted odds ratios in Nyanza, Kenya

Individual variables for both analyses	Experienced diarrhea within the previous 2 weeks			Appropriate treatment		
	No n (%)	Yes n (%)	Unadjusted odds ratio	Inappropriate n (%)	Appropriate n (%)	Unadjusted odds ratio
Child’s gender						
Female	2102 (86.1)	339 (13.9)	Ref.	213 (64.0)	120 (36.0)	Ref.
Male	2072 (82.4)	442 (17.6)	1.390***	268 (61.1)	171 (39.0)	1.058
Child’s age (years)						
< 1	767 (79.2)	202 (20.9)	Ref.	126 (62.4)	76 (37.6)	Ref.
1	647 (75.0)	216 (25.0)	1.259*	124 (58.2)	89 (41.8)	1.132
2	849 (83.5)	168 (16.5)	0.722**	109 (66.1)	56 (33.9)	0.768
3	984 (90.0)	109 (10.0)	0.375***	64 (59.3)	44 (40.7)	1.131
4	927 (91.5)	86 (8.5)	0.350***	58 (69.1)	26 (31.0)	0.601
Relationship to household head						
Biological child	3294 (84.2)	618 (15.8)	Ref.	386 (63.1)	226 (36.9)	Ref.
Grandchild	745 (85.4)	127 (14.6)	0.840	74 (59.7)	50 (40.3)	1.158
Other	135 (79.0)	36 (21.1)	1.376	21 (58.3)	15 (41.7)	1.123
Household head’s gender						
Female	1,039 (84.1)	197 (15.9)	Ref.	118 (61.8)	73 (38.2)	Ref.
Male	3,135 (84.3)	584 (15.7)	1.030	363 (62.5)	218 (37.5)	1.019
Age group (years)						
< 30	1101 (80.4)	269 (19.6)	Ref.	172 (64.9)	93 (35.1)	Ref.
30–34	788 (84.4)	146 (15.6)	0.767***	87 (59.6)	59 (40.4)	1.276
35–39	700 (86.0)	114 (14.0)	0.642***	67 (59.8)	45 (40.2)	1.433
40–49	750 (87.4)	108 (12.6)	0.612***	66 (62.9)	39 (37.1)	1.034
≥ 50	835 (85.3)	144 (14.7)	0.681***	89 (61.8)	55 (38.2)	1.147
Education level						
No education/Primary level	2829 (83.5)	561 (16.6)	Ref.	342 (62.0)	210 (38.0)	Ref.
Secondary level or higher	1345 (85.9)	220 (14.1)	0.862	139 (63.2)	81 (36.8)	0.913
Household wealth index						
Poorer	985 (84.0)	188 (16.0)	Ref.	123 (67.2)	60 (32.8)	Ref.
Poor	868 (83.7)	169 (16.3)	1.054	105 (62.5)	63 (37.5)	1.174
Middle	855 (85.0)	151 (15.0)	0.960	85 (56.3)	66 (43.7)	1.644*
Rich	802 (85.6)	135 (14.4)	0.906	80 (60.6)	52 (39.4)	1.377
Richer	664 (82.8)	138 (17.2)	1.062	88 (63.8)	50 (36.2)	1.075
Number of household members						
5 or fewer than 5	2002 (83.2)	404 (16.8)	Ref.	253 (63.3)	147 (36.8)	Ref.
More than 5	2172 (85.2)	377 (14.8)	0.824*	228 (61.3)	144 (38.7)	1.111
Number of children under five						
One	1340 (86.3)	213 (13.7)	Ref.	135 (64.6)	74 (35.4)	Ref.
More than 1	2834 (83.3)	568 (16.7)	1.203*	346 (61.5)	217 (38.5)	1.190
Paying attention to the child						
Less attention	2316 (83.3)	464 (16.7)	Ref.	278 (60.4)	182 (39.6)	Ref.
More attention	1858 (85.4)	317 (14.6)	0.844*	203 (65.1)	109 (34.9)	0.892
Playing with the child in the past three days						
No playing	858 (84.8)	154 (15.2)	Ref.	102 (68.5)	47 (31.5)	Ref.
Playing at least once within the past 3 days	3316 (84.1)	627 (15.9)	1.028	379 (60.8)	244 (39.2)	1.233
Individual Variables for the first analysis on diarrhea cases						
Water source for drinking						
Unimproved	2057 (84.1)	388 (15.9)	Ref.			
Improved	2117 (84.3)	393 (15.7)	0.974			
Water treatment						
No treatment/Inadequate	1839 (83.0)	378 (17.1)	Ref.			
Adequate treatment	2335 (85.3)	403 (14.7)	0.820*			
Hand-washing facility						
Not available	4045 (84.1)	764 (15.9)	Ref.			
Available	129 (88.4)	17 (11.6)	0.620			
Possession of refrigerator						
No	4096 (84.1)	772 (15.9)	Ref.			
Yes	78 (89.7)	9 (10.3)	0.491			
Individual Variables for the second analysis on appropriate treatment						
Severity of diarrhea						
Without blood				437 (64.9)	236 (35.1)	Ref.
With blood				44 (44.4)	55 (55.6)	2.538***
Possession of at least one media device						
No				47 (58.8)	33 (41.3)	Ref.
Yes				434 (62.7)	258 (37.3)	0.809
Community Variables for the two analyses						
Area						
Urban	402 (79.6)	103 (20.4)	Ref.	66 (65.4)	35 (34.7)	Ref.
Rural	3772 (84.8)	678 (15.2)	0.753*	415 (61.9)	256 (38.2)	1.195
Performance of CHWs in the CU						
None/Poor (Number of CHWs and CUs)	2263 (85.8)	376 (14.3)	Ref.	248 (67.0)	122 (33.0)	Ref.
Middle	1244 (83.9)	239 (16.1)	1.194	141 (59.5)	96 (40.5)	1.651**
High	667 (80.1)	166 (19.9)	1.453**	92 (55.8)	73 (44.2)	1.841**

* ≤ 0.05 ** ≤ 0.01 *** ≤ 0.001

Table 3 presents the factors associated with the prevalence of childhood diarrhea in Nyanza Province, Kenya. According to the likelihood ratio test, we concluded that model 2 was the final model. In model 2, the increasing age of the child was negatively associated with the prevalence of childhood diarrhea (AOR: 0.714; 95%CI: 0.669–0.761; *p* < 0.001), while male children were significantly more likely to have diarrheal diseases (AOR: 1.429; 95%CI: 1.192–1.714; *p* < 0.001). Compared with households where the head was under 30 years old, children staying with household heads aged 35–39 years old were significantly less likely to have childhood diarrhea (AOR: 0.718; 95%CI: 0.534–0.967; *p* = 0.029). A child being paid more attention was less likely to develop diarrheal disease (AOR: 0.693; 95%CI: 0.579–0.828; *p* < 0.001). In addition, treating water (AOR: 0.799; 95%CI: 0.651–0.980; *p* = 0.031) was significantly associated with the prevention of diarrhea. Children living in rural areas were significantly less likely to experience diarrhea symptoms than those living in urban areas (AOR: 0.666; 95%CI: 0.455–0.996; *p* = 0.048). Areas with high CU performance were positively associated with the prevalence of diarrhea, compared with areas without CUs or with poor performing CUs (AOR: 1.347; 95%CI: 1.003–1.810; *p* = 0.048).

**Table 3:** Multilevel logistic regressions predicting factors associated with prevalence of diarrhea among children younger than five years of age in Nyanza Province, Kenya

Individual variables	Model 1		Model 2	
	Odds ratio	95% CI	Odds ratio	95% CI
Child’s gender				
Female	Ref.		Ref.	
Male	1.424***	1.189–1.706	1.429***	1.192–1.714
Child’s age (continuous from 0 to 4)	0.712***	0.668–0.759	0.714***	0.669–0.761
Relationship to household head				
Biological child	Ref.		Ref.	
Grandchild	0.979	0.672–1.427	0.992	0.681–1.446
Other	1.508	0.976–2.328	1.505	0.978–2.317
Household head’s gender				
Female	Ref.		Ref.	
Male	1.003	0.810–1.241	1.001	0.808–1.240
Age group (years)				
< 30	Ref.		Ref.	
30–34	0.826	0.624–1.094	0.833	0.630–1.100
35–39	0.719*	0.534–0.968	0.718*	0.534–0.967
40–49	0.729	0.517–1.029	0.738	0.523–1.041
≥ 50	0.803	0.542–1.189	0.814	0.550–1.204
Education level				
No education/Primary level	Ref.		Ref.	
Secondary level or higher	0.895	0.727–1.101	0.911	0.740–1.121
Household wealth index				
Poorer	Ref.		Ref.	
Poor	1.148	0.874–1.509	1.134	0.862–1.491
Middle	0.964	0.723–1.285	0.946	0.709–1.264
Rich	0.906	0.676–1.214	0.858	0.637–1.155
Richer	1.237	0.885–1.730	1.045	0.723–1.510
Number of household members				
5 or fewer than 5	Ref.		Ref.	
More than 5	0.933	0.748–1.165	0.949	0.759–1.186
Number of children under five				
One	Ref.			
More than 1	1.159	0.951–1.413	1.154	0.946–1.407
Paying attention to the child				
Less attention	Ref.			
More attention	0.690***	0.577–0.824	0.693***	0.579–0.828
Playing with the child in the past three days				
No playing	Ref.		Ref.	
Playing at least once within the past 3 days	1.084	0.844–1.391	1.076	0.838–1.381
Water source for drinking				
Unimproved	Ref.		Ref.	
Improved	0.956	0.775–1.179	0.941	0.761–1.164
Water treatment				
No treatment/Inadequate	Ref.		Ref.	
Adequate treatment	0.819	0.669–1.004	0.799*	0.651–0.980
Hand-washing facility				
Not available	Ref.		Ref.	
Available	0.753	0.378–1.500	0.741	0.368–1.492
Possession of refrigerator				
No	Ref.		Ref.	
Yes	0.649	0.290–1.451	0.611	0.266–1.403
Community Variables				
Area				
Urban			Ref.	
Rural			0.666*	0.445–0.996
Performance of CHWs in the CU				
None/Poor			Ref.	0.941–1.487
Middle			1.183	1.003–1.810
High			1.347*	
Community Level Variance and covariance of random effects	0.294 (0.065)		0.262 (0.061)	
Log likelihood	-2038.89		-2033.98	
Likelihood-ratio test (*p* value)	≤0.001		0.019	

* ≤ 0.05 *** ≤ 0.001

### Determinants of appropriate treatment for childhood diarrhea

The results of a bivariate analysis are shown in Table 2. According to this analysis, the factors significantly associated with appropriate treatment were household wealth index, severity of diarrhea and the performance of the CUs. These were the same significant factors in the multilevel analysis below.

Table 4 shows the determinants of appropriate treatment for childhood diarrhea. Among individual variables in model 2, household wealth and severity of diarrhea were significantly associated with an appropriate treatment. Compared with people in the lowest wealth quintile, children of families in the middle wealth quintile had around twice the odds of receiving the necessary health care (AOR: 1.923; 95%CI: 1.018–3.631; *p* = 0.044). Childhood diarrhea with blood was also significantly associated with an appropriate treatment (AOR: 2.813; 95%CI: 1.508–5.251; *p* = 0.001). Compared with areas without CUs or with lower performing CUs, the areas with middle (AOR: 1.655; 95%CI: 1.080–2.538; *p* = 0.021) and higher performing (AOR: 1.938; 95%CI: 1.201–3.127; *p* = 0.007) CUs had significantly increased odds of practicing appropriate treatment.

**Table 4:** Multilevel logistic regressions predicting factors associated with appropriate treatment for childhood diarrhea in Nyanza Province, Kenya

Individual variables	Model 1		Model 2	
	Odds ratio	95% CI	Odds ratio	95% CI
Child’s gender				
Female	Ref.		Ref.	
Male	1.013	0.693–1.482	0.998	0.684–1.456
Child’s age (continuous from 0 to 4)	0.883	0.772–1.008	0.889	0.780–1.013
Relationship to household head				
Biological child	Ref.		Ref.	
Grandchild	1.660	0.814–3.386	1.758	0.864–3.577
Other	1.462	0.614–3.481	1.369	0.578–3.242
Household head’s gender				
Female	Ref.		Ref.	
Male	1.125	0.706–1.792	1.118	0.708–1.764
Age group (years)				
< 30	Ref.	Ref.	Ref.	
30–34	1.385	0.788–2.431	1.374	0.796–2.372
35–39	1.358	0.721–2.557	1.325	0.711–2.467
40–49	1.062	0.535–2.107	1.005	0.510–1.984
≥ 50	0.809	0.372–1.760	0.752	0.346–1.633
Education level				
No education/Primary level	Ref.		Ref.	
Secondary level or higher	0.972	0.636–1.485	0.989	0.648–1.509
Household wealth index				
Poorer	Ref.		Ref.	
Poor	1.506	0.838–2.704	1.475	0.836–2.603
Middle	1.937*	1.018–3.686	1.923*	1.018–3.631
Rich	1.663	0.912–3.032	1.587	0.867–2.905
Richer	1.598	0.833–3.067	1.594	0.760–3.344
Number of household members				
5 or fewer than 5	Ref.		Ref.	
More than 5	0.968	0.615–1.525	1.011	0.642–1.591
Number of children under five				
One	Ref.		Ref.	
More than 1	1.212	0.790–1.859	1.193	0.781–1.823
Paying attention to the child				
Less attention	Ref.		Ref.	
More attention	0.809	0.557–1.177	0.815	0.564–1.178
Playing with the child in the past three days				
No playing	Ref.		Ref.	
Playing at least once within the past 3 days	1.228	0.727–2.076	1.205	0.717–2.027
Severity of diarrhea				
Without blood	Ref.		Ref.	
With blood	2.821***	1.499–5.311	2.813***	1.508–5.251
Possession of at least one media device				
No	Ref.		Ref.	
Yes	0.641	0.338–1.216	0.650	.347–1.217
Community Variables				
Area				
Urban			Ref.	
Rural			1.117	0.551–2.265
Performance of CHWs				
None/Poor			Ref.	
Middle			1.655*	1.080–2.538
High			1.938**	1.201–3.127
Community Level Variance	0.516 (0.224)		0.402	
Log likelihood	-484.54		-479	
Likelihood-ratio test (*p* value)	0.030		0.027	

* ≤ 0.05 ** ≤ 0.01 *** ≤ 0.001

## Discussion

This study aims to evaluate the association between the performance of CUs and the prevalence of diarrhea among children under five in Nyanza Province, Kenya, and the appropriate treatment for childhood diarrhea. Compared with the Kenya Demographic Health Survey (KDHS) [9], the percentage of diarrhea cases among children under five was similar: 16.6% in KDHS and 15.8% in this study. Since the burden of diarrheal diseases is still substantial, it is clear that more action is needed to reduce the incidence of diarrhea. In addition, people in the community must be encouraged to take appropriate action regarding their children’s diarrhea. The following significant factors associated with diarrhea prevalence and the appropriate treatments for childhood diarrhea could be useful in developing an effective strategy for both prevention and treatment.

### Factors associated with childhood diarrhea

In our study, the significant factors negatively associated with childhood diarrhoea were the child’s increasing age, the household head being in the middle age group, the child being paid more attention, treated drinking water and residence in a rural area, while male children and those in areas with high performing CUs were significantly more likely to have childhood diarrhea.

The areas with high performing CUs were associated with more diarrheal diseases. This might be because CU establishments were focused more on areas with a high prevalence of diarrhea and other diseases. According to the community health services focal officers at the research sites, the officers are responsible for prioritizing areas that require urgent addressing of health needs, especially childhood diseases, and behavior that leads to poor health by community members, because the Ministry of Health was unable to establish all CUs at the same time. Prioritized areas were therefore chosen for the establishment of CUs and allocation of more resources. Other studies have shown that routine visitation by CHWs was effective in reducing the incidence of childhood diarrhea [14–16]. The study in Kenya also shows that CU establishment can increase water treatment and latrine use [10], major risk factors of childhood diarrhea. According to the additional bivariate analysis, our data also suggested the same relationship: the areas covered by high performance CUs had a higher percentage of water treatment (*p* < 0.001). It also suggested that CHWs could have a positive impact on the use of water treatment, which may reduce the incidence of childhood diarrhea in the near future.

In this study, children living in rural area were less likely to have experienced diarrhea than children in urban areas. One possible reason for this is that research conducted in Kenya shows that memories among people in rural areas fade more easily than those of urban residents. This means that people in urban areas seem to remember such events well [17]. People living in urban areas might be more cognizant of their children’s symptoms because of more information available in urban areas; this would therefore improve their health awareness. In addition, population density is an important determinant influencing risk of disease transmission [18, 19]. This would be the reason why the diarrhea cases in urban areas, which would be the areas with higher population densities, were higher than rural.

The incidence of diarrhea among children under 1 and aged 1–2 years was 20.9% and 25% respectively, while the percentage among children aged 4–5 years was 8.5%. Children under two years old in this study were the most vulnerable in terms of the diarrheal infection. Another study also shows that the young age of the child, especially after exclusive breastfeeding, is linked to a greater risk of having diarrhea [20, 21]. Appropriate infant feeding following exclusive breastfeeding is an important intervention for the reduction of childhood diarrhea and improvement of the child’s health [22–25].

If children were left alone in the house or only with other siblings under 10 years of age, their parents would by implication pay less attention to those children than others. As a result, children without sufficient attention are more likely to have accidents or behave dangerously, such as eating dirty food from the floor [11]. Furthermore, parents who did not stay with their children may work outside of the house. Parental employment is reported as a significant factor in childhood diarrhea [26]; it is also reported that women’s participation in income generating activities has positive effect on their children’s nutritional condition [22]. Further research is needed to clarify details regarding children who are left alone and the effects of this.

In addition, the effectiveness of water treatment was reported by other researchers as an important factor in preventing childhood diarrhea [27–31]. Our study also suggested that water treatment practices are recommended interventions for the prevention of childhood diarrhea.

### Determinants of appropriate treatment for childhood diarrhea

Although we included a range of variables in the analysis to examine significant factors for the appropriate treatment of childhood diarrhea, household wealth index, severity of diarrhea and the middle and high performance of CUs were the only significant variables in this study. A study in Kenya by Olson et al demonstrates how CHWs are important health personnel who could play a role in delivering health-related information [32]. Colvin [33] also reported that a middle layer between the community and a health facility, such as the CHWs, is an important element in influencing appropriate care-seeking behavior. In addition, other studies describe the effectiveness of CHWs in promoting the uptake of appropriate treatment for childhood diarrhea [34]. A wider implementation of CHWs, through CU establishments, would be the effective means of improving the accessibility of health care, regardless of individual factors. Although a study has been done that assesses the influencing factors of CHWs’ performance [5, 35], it is important to conduct further research on interventions to increase and sustain the high performance of CHWs and CUs.

One key reason for not seeking care is cost of treatment [36]. It is also reported that household wealth was a determinant of health seeking behavior for childhood diarrhea [37]. The trend that individuals from poorer households tend to be less likely to seek health services is also reported in studies of other curative [38] and preventive services, such as immunization [39]. In these studies, we see that the caregivers in wealthier households could afford to pay not only the cost of health services but also transportation and other costs.

As this study identified, severity of diarrhea is an important factor associated with appropriate treatment. Lack of maternal perception on the seriousness of the illness was shown to be the primary reason for no treatment [36]. In addition, perception of severity of illness is associated with appropriate health seeking behavior, such as health facility visitation [40]. Bloody diarrhea would make caregivers think seriously about their child’s diarrhea and encourage them to take necessary actions.

### Limitations

This present study has reported the significant factors, including the performance of CUs, associated with diarrhea prevalence and appropriate treatment for diarrhea among children under five in western Kenya.

However, this study is a cross-sectional study. Since the effects of CUs could change over time, and there is a possibility that areas with a high prevalence of diarrhea were selected when establishing the CUs, further longitudinal research is necessary to clarify the causal connection between CHW performance, diarrhea prevalence and appropriate treatment. The evaluation framework of the CUs’ performance is another limitation. Although we utilized the official evaluation framework developed by the Kenyan MoH, the CU performance markers used in this study related to group level performance, which is not equivalent to individual performance. Therefore, our study shows only the association between group performance, prevalence of childhood diarrhea and appropriate treatment. Further research on the effects of CHWs needs to be conducted by using an evaluation framework that includes both personal and group performance indicators, such as number of households visited per month, job satisfaction and health knowledge, as well as evaluations from supervisors and community members. Since two of the three performance indicators derived from documentation, there is also a possibility that although the meetings were actually held, CUs did not submit minutes of meetings and were regarded as poorly performing. It would be better for further research to integrate indicators such as number of households visited into the performance evaluation framework. In addition, the causal relations among CU performance, household practices to prevent childhood diarrhea and uptake of appropriate treatment were unclear.

Since the MICS data was collected using a structured questionnaire and not direct observation, several variables, such as the severity of diarrhea, means of water treatment and drinking water resource, may not be accurate. Although this study assumed that household heads would be the most influential people in decision making, there is a possibility that mothers or other family members have autonomy to make decisions about care-seeking for children in their households.

Furthermore, periods of time for two variables, child left alone more than once and playing with the child, were not measured in this study. It would be better to assess the period of each activity and make their effects clearer.

## Conclusion

Our study has clarified several factors significantly associated with diarrhea prevalence and appropriate treatment for diarrhea for children under five in Nyanza Province, Kenya. In this study, we addressed a significant factor for both outcomes: the performance of CUs, represented by the performance of CHWs, CHC and CHEWs. Although their performance was significantly associated with the higher prevalence of childhood diarrhea, the areas with a high prevalence of diarrhea would have been primarily selected to establish CUs. However, higher CU performance was also significantly associated with taking an appropriate treatment for childhood diarrhea. It is suggested that high performance of CU might have a positive effect on encouraging community members to select appropriate treatment for childhood diarrhea. With full consideration of the evaluation framework, further studies are needed in order to describe the causal connection between CHW performance and diarrhea prevention and treatment, as well as for better understanding of long-term impacts.

## Abbreviations

CHC: Community health committee
CHEW: Community health extension worker
CHIS: Community health information system
CHS: Community health strategy
CHWs: Community health workers
CU: Community unit
EAs: Enumeration areas
KDHS: Kenya demographic health survey
KNBS: Kenya National Bureau of Statistics
MICS: Multiple indicator cluster survey
MoH: Ministry of health
MoPHS: Ministry of public health and sanitation
NHPP II: National health sector strategic plan 2005–2010
PPS: Probability proportional to size
